# Influence of Dopant Nature on Biological Properties of ZnO Thin-Film Coatings on Ti Alloy Substrate

**DOI:** 10.3390/nano10010129

**Published:** 2020-01-10

**Authors:** Stefania Stoleriu, Codruta Lungu, Cristina Daniela Ghitulica, Adrian Surdu, Georgeta Voicu, Andreia Cucuruz, Claudiu Stefan Turculet, Lucian Toma Ciocan

**Affiliations:** 1 Department of Science and Engineering of Oxide Materials and Nanomaterials, University Politehnica of Bucharest, 1-7 Gh. Polizu Street, RO-060041 Bucharest, Romania; stefania.stoleriu@upb.ro (S.S.); cristina.ghitulica@upb.ro (C.D.G.); adrian.surdu@upb.ro (A.S.); 2 Department of Biomaterials and Medical Devices, University Politehnica of Bucharest, 1-7 Gh. Polizu Street, RO-011061 Bucharest, Romania; codruta.lungu@icloud.com; 3 Department of Surgery, “Carol Davila” University of Medicine and Pharmacy, 8 Eroii Sanitari Street, RO-050474 Bucharest, Romania; 4 Department of Prosthetics Technology and Dental Materials, “Carol Davila” University of Medicine and Pharmacy, 8 Eroii Sanitari Street, RO-050474 Bucharest, Romania; tciocan@yahoo.com

**Keywords:** ZnO thin film, spin coating, antibacterial test, *in-vitro* test

## Abstract

In this paper, ZnO and Co^2+^/Mg^2+^-doped ZnO thin films on TiAlV alloy substrates were obtained. The films were deposited by spin coating of sol-gel precursor solutions and thermally treated at 600 °C for 2 h, in air and slow cooled. The doping ions concentration was 1.0 mol%. The study’s aim was to obtain implantable metallic materials with improved biocompatibility and antibacterial qualities. The characteristics of the thin films were assessed from the point of view of microstructure, morphology, wetting properties, antibacterial activity and biological response in the presence of amniotic fluid stem cells (AFSC). The results proved that all deposited samples were nanostructured, suggesting a very good antibacterial effect and proving to be suitable supports for cellular adhesion and proliferation. All properties also depended on the doping ion nature.

## 1. Introduction

Microbial infectious diseases are caused by pathogenic microorganisms, such as bacteria, viruses, parasites or fungi, and are a major cause of morbidity and mortality [1,2]. In the last decade, there has been an increase in the number of pathogens that confer multiple resistance to drugs and to various antibacterial agents. Several types of strains, such as *Escherichia coli, Klebsiella pneumoniae*, *Enterobacteriaceae*, *Pseudomonas aeruginosa*, *Staphylococcus aureus* and *Enterococcus* have been recognized as the pathogens that have developed the highest resistance to conventional remedies [3]. The ideal solution to combat these increasingly powerful pathogens is to develop new classes of antimicrobial agents [4,5,6].

An antibacterial agent used against infectious diseases could be ZnO that has been extensively studied by many researchers owing to its potential applications in for example gas sensors, photo-catalysts, dye-sensitized solar cells, antibacterial applications, water purification, textiles, food packaging, pharmaceutical and biological applications. Zinc oxide nanoparticles are a semiconductor material with a wide band gap (~3.37 eV), large exciton-binding energy (~60 meV) and chemical stability [7,8,9]. The mechanism of antibacterial action of the material is that the production of reactive oxygen species on the surface of these ZnO nanoparticles in light causes oxidative stress in bacterial cells and eventually leads to the death of the cells. Reactive oxygen species contain the most reactive hydroxyl radical, a less toxic superoxide anion radical, and hydrogen peroxide with a weaker oxidizer [10].

To enhance its performance, significant efforts have been made, i.e., doping ions, aiming to increase the defects in the lattice and a higher generation of e^−^/h^+^ pairs and a reduction in its band-gap energy. ZnO doping is very important because the doping ions can effectively modify the structural and morphological properties, as well as the electrical, optical and magnetic properties, which can confer these nanomaterials on the basis of ZnO properties enabling their inclusion in applications in different fields [1,11,12].

Until now, several attempts have been conducted on doping metal ions (Al, Ca, Ce, Co, Cu, Fe, Mn, Mg) in the ZnO lattice [7,13,14,15,16,17,18,19,20]. Among the aforementioned, magnesium-doped ZnO exhibited better antibacterial activity because Mg^2+^ (0.72 Å) can substitute Zn^2+^ (0.74 Å) sites owing to their similar ionic radii, which induces more reactive species generation from the nanoparticles’ surface. MgO is an ionic semiconductor compound with a broad band gap of 7.8 eV and the incorporation of Mg^2+^ in ZnO host lattice improves the optical characteristics of ZnO [21,22,23].

Dopants like Mn^2+^ or Co^2+^ are important ions as they are optically active and provide localized spins which interact with the spins of electrons and holes in the host lattice and can be used in applications such as spin-valve transistors, spin-light emitting diodes and non-volatile storage. The cobalt doped ZnO thin films have various applications such as transparent conductive, ferromagnetism, semiconductors, piezoelectric and solar cells. As well, doping with Co ions favours the antibacterial activity [12,24].

In the last few years, there has been a growing interest in the development of antimicrobial coatings based on thin films [2] and ZnO is an important candidate material for thin film [25]. Also, to obtain doped ZnO, the literature data suggests obtaining different experimental results in correlation with the synthesis method. Deposition methods were employed for the preparation of doped ZnO thin films such as spray pyrolysis, sol-gel spin coating, chemical bath deposition, electrodeposition and electron beam evaporation. These thin films may present several properties such as optoelectrical and band-gap energy, different chemical compositions (stoichiometries), and micro-structures, like the crystalline domains size (grain size), morphology, roughness and porosity. Due to their unique properties, the thin films of ZnO have possible applications in various areas of science and technology [2,21,26].

## 2. Materials and Methods

### 2.1. Materials and Synthesis of the Samples

Undoped and Co^2+^/Mg^2+^-doped ZnO thin films were deposited on grade TiAlV alloy substrates by the spin coating technique. The sol-gel precursor solutions used were obtained from zinc acetate dihydrate (Zn(CH_3_COO)_2_·2H_2_O, ≥99%, Sigma-Aldrich, Saint Luis, MI, USA), cobalt sulphate heptahydrate (CoSO_4_·7H_2_O, ≥99%, Sigma-Aldrich, Saint Luis, MI, USA) or magnesium acetate tetrahydrate (Mg(CH_3_COO)_2_·4H_2_O, ≥99%, Sigma-Aldrich, Saint Luis, MI, USA) as cation precursors, monoethanolamine, MEA (H_2_NCH_2_CH_3_OH, ≥98%, Sigma-Aldrich, Saint Luis, MI, USA) was employed as synthesis additive for pH adjustment, as well as absolute ethanol (CH_3_CH_2_OH, Sigma-Aldrich, Saint Luis, MI, USA) as a solvent.

The concentration of doping ions (Co^2+^ or Mg^2+^) was 1 mol% and the molar ratio between Zn(CH_3_COO)_2_·2H_2_O and MEA was set at 1:2. The salts of cobalt, magnesium and zinc, the solvent and MEA were mixed and homogenized by magnetic stirring at 60 °C for 2 h; after that the water necessary for hydrolysis was added; the solution was mixed for one minute. The colloids obtained had the different colours: the undoped system and Mg^2+^ doped were opaque white, while the one containing Co^2+^ doped was light purple. One thin film was composed of 3 layers; layer deposition was performed immediately after the hydrolysis (water addition), before beginning the gelification process. This was made by spinning 3 drops of precursor colloids at a rotation speed of 1500 rpm, clockwise for a rotation time of 1 min. The sequence was repeated 3 times in order to obtain two-dimensional structures composed of 3 layers. After each layer, the sample was dried at 150 °C for 5 min to remove most of the solvent using a thermostat hot plate. The deposited samples were kept at 60 °C for 24 h in an oven for soft drying and maturation. After drying, the samples were thermally treated at 600 °C for 2 h, in air, with a heating rate of 5 °C/min and slow cooled.

### 2.2. Characterization

The coatings obtained were characterised from the point of view of phase composition, microstructural characteristics and biological behaviour.

The crystalline phase composition of coatings was assessed by X-ray diffraction (XRD) measurements at room temperature, using a Panalytical Empyrean diffractometer (step size 0.02°; time per step 1°/s), with Ni-filtered Cu Kα radiation (λ = 0.154 nm), 2θ ranging between 10 and 80°. Also, the average crystallite size was calculated from XRD results using the Debye–Scherrer equation.

To obtain detailed information on the constituent phases’ polymorphism, Raman spectroscopy was used by employing a Horiba LabRAM HR Evolution spectrometer (with Ar laser (λ = 514 nm), acquisition time 180 s and the result was an arithmetic average of three measurements.

The microstructural evaluation was performed using a FEI Quanta Inspect F50 scanning electron microscope (SEM, 1.2 nm resolution), coupled with energy-dispersive X-ray diffraction analysis (EDX).

The biological behaviour was assessed by: (i) the hydrophilic/hydrophobic balance (measuring water static contact angle); (ii) the antibacterial test (conducted on *Staphylococcus aureus*, a Gram-positive bacteria) [27]; (iii) the in vitro test (assays of cell viability and proliferation: MTT assay [28]—a colorimetric method for estimating the cellular metabolic activity; glutathione (GSH) assay [29]—a luminescence method for predicting the toxicological response).

(i) The hydrophilic/hydrophobic balance of coatings was assessed by measuring the static contact angle between a drop of distilled water and the deposition surface at room temperature. The contact angle (θ) was measured used a contact Angle Meter–KSV Instruments CAM 100 equipment. Each experiment was conducted in triplicate and the mean values are presented. The surface is hydrophilic if contact angle is between 0–90°, and it was hydrophobic if the contact angle was over 90°. Also, the surface free energy was determined based on θ value, using Equation (1):

ξ = γ·cosϴ(1)

where: ξ- surface free energy; θ-contact angle; γ-water superficial tension (72.8 mN/m).

For the present study we used only one polar liquid, i.e., water (based on the fact that the simulated body fluid is an aqueous solution). Considering this, the mathematical model used for the surface free energy calculation is a simple model [30]. The value for surface free energy was calculated using the mean values of contact angle.

(ii) The antibacterial assay was assessed by determination of minimal inhibitory concentration (MIC) of the ZnO coatings.

For determination of MIC, a strain of *Staphylococcus aureus (S. a)* ATCC 25,923 was purchased from the American Type Culture Collection (Virginia, USA) and maintained in the Lab as Glycerol stocks. Fresh cultures obtained in nutritive broth from glycerol stocks were inoculated on LB agar (for bacteria) and incubated for 24 h at 37 °C to obtain cultures that were used for all subsequent studies. The antibacterial activity of various zinc oxide depositions has been studied by determining the minimum inhibitory concentration against a *S. aureus* laboratory strain. The samples were sterilized by ultraviolet (UV) treatment for 20 min. Microbial suspensions prepared in sterile saline buffer were obtained from each strain and adjusted to an optical density of 0.5 McFarland (1.5 × 108 CFU (colony forming units)/mL). These were used to inoculate the entire surface of the nutrient agar Petri dishes. For assessing monospecific biofilm formation, 2 mL of nutritive broth were disposed in each well of a 6-well plate, containing test (coated TiAlV substrate) and control (TiAlV substrate) samples and seeded with the bacterial inoculum consisting of a volume of 50 μL from the phosphate-buffered saline (PBS) bacterial suspension. After a period of 24 h incubation at 37 °C, the materials containing attached bacteria, were washed with PBS and transferred to a fresh well containing 2 mL sterile nutritive broth, and the incubation continued for another 24 h. After that, biofilm-embedded bacterial cells were detached by vigorous vortexing for 30 s. PBS suspensions containing detached bacteria cells were subjected to serial 10-fold dilutions and each dilution was seeded on nutritive agar. Experiments were performed in triplicate and repeated on at least three separate occasions. Also, the MIC was considered as the lowest concentration to inhibit the microbial development. This was established through evaluation by the naked eye and spectroscopy measurement of microbial cultures at Abs = 600 nm.

(iii) In vitro evaluation of biocompatibility by MTT assay is a quantitative colorimetric method, which allows evaluation of cell viability and proliferation, and cytotoxicity of different samples: the TiAlV substate and ZnO depositions. The method is based on reduction of MTT tetrazolium salt (3-(4,5-dimethylthiazolyl)-2,5-diphenyltetrazolium bromide) to dark blue formazan. Reduction by mitochondrial enzymes (especially succinate dehydrogenase) is an indication of cell/mitochondrial integrity. Formazan, insoluble in water, can be solubilized with isopropanol, dimethylsulfoxide or other organic solvent. The optical density (OD) of solubilized formazan is evaluated spectrophotometrically, resulting in a colour-absorbent–colour-counting function of the number of metabolic active cells in the culture.

Human mesenchymal amniotic fluid stem cells (AFSC) were used to evaluate the biocompatibility of ZnO depositions. The cells were cultured in Dulbecco’s modified Eagle’s medium (DMEM, Sigma-Aldrich, Saint Luis, MI, USA) supplemented with 10% foetal bovine serum, 1% penicillin and 1% streptomycin antibiotics (Sigma-Aldrich, Saint Luis, MI, USA). To maintain optimal culture conditions, the medium was changed twice a week. The biocompatibility was assessed using MTT assay (Vybrant^®^ MTT Cell Proliferation Assay Kit, Thermo Fischer Scientific, Waltham, MA, USA). Briefly, the AFSC were grown in 96-well plates, with a seeding density of 3000 cells/well in the presence of samples for 72 h. Then 10 µL Solution I (12 mM MTT) was added and incubated at 37 °C for 4 h. Then, 100 µL of Solution II (1 mg sodium dodecyl sulphate + 10 mL HCl, 0.01 M) was added and pipetted vigorously to solubilize formazan crystals. After 1 h the absorbance was read using a spectrophotometer at 570 nm (TECAN Infinite M200, Männedorf, Switzerland).

The GSH-Glo assay (GSH-Glo^TM^ Glutathione Assay, Promega, Madison, WI, USA) is a luminescent-based assay for the detection and quantification of glutathione (GSH) in cells or in various biological samples. A change in GSH levels is important in the assessment of toxicological responses and is an indicator of oxidative stress, potentially leading to apoptosis or cell death. The assay is based on the conversion of a luciferin derivative into luciferin in the presence of GSH. The reaction is catalysed by a glutathione S-transferase (GST) enzyme supplied in the kit. The luciferin formed is detected in a coupled reaction using Ultra-Glo Recombinant Luciferase that generates a glow-type luminescence that is proportional to the amount of glutathione present in cells. The assay provides a simple, fast and sensitive alternative to colorimetric and fluorescent methods and can be adapted easily to high-throughput applications.

AFSC were seeded at a density of 3000 cells in 300 µL of DMEM supplemented with 10% foetal bovine serum and 1% antibiotics (penicillin, streptomycin/neomycin) in 96 well plates. After seeding 24 h, cells were treated with samples and incubated for 72 h.

The working protocol consisted of adding 100 µL 1X GSH-Glo Reagent and incubating at 37 °C for 30 min. Then, 100 µL Luciferin Deection Reagent was added and incubated at 37 °C for an additional 15 min. At the end of the time, the wells were well homogenized and then the plate was read on the luminometer (MicroplateLuminometerCentro LB 960, Berthold, Germany).

The biocompatibility of the TiAlV substrate and ZnO depositions was also evaluated by fluorescence microscopy, using RED CMTPX fluorophore (Thermo Fischer Scientific, Waltham, MA, USA), a cell tracker for long-term tracing of living cells. The CMTPX tracker was added in cell culture treated with samples and the viability and morphology of the AFSC was evaluated after 5 days. The CMTPX fluorophore was added in the culture medium at a final concentration of 5 µM, incubated for 30 min in order to allow dye penetration into the cells. Next, the AFSC were washed with PBS and visualized by fluorescent microscopy. The photomicrographs were taken with an Olympus CKX 41 digital camera driven by CellSense Entry software (Olympus, Tokyo, Japan).

## 3. Results and Discussion

### 3.1. ZnO and Co^2+^/Mg^2+^ Doped ZnO Coatings Morphology and Phase Composition

**Figure 1:**
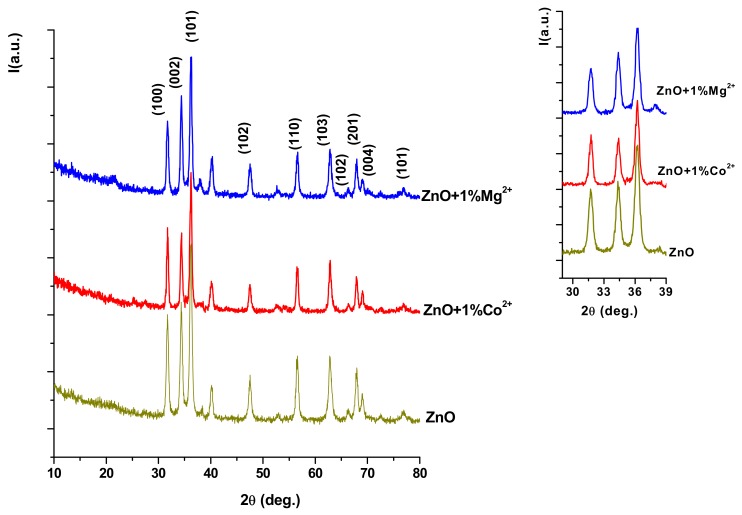
X-ray diffraction (XRD) patterns for undoped and Co^2+^/Mg^2+^doped ZnO films coatings on grade TiAlV alloy thermally treated at 600 °C for 2 h.

Figure 1 shows the XRD patterns of the undoped and Co^2+^/Mg^2+^-doped ZnO films coatings obtained at 600 °C for 2 h. One can observe the characteristic peaks of wurtzite (ASTM 80-0075), indifferent of the deposition nature (undoped or Co^2+^/Mg^2+^doped ZnO). Also, two low-intensity peaks between 38–42° are attributed to titanium substrate (ASTM 89-2959; 44-1294) and to a non-stoichiometric titanium oxide (ASTM 80-2540).

The doping of ZnO with Co^2+^/Mg^2+^modifies the ratio between the intensities of principal peaks ((100), (002) and (101)), and this can be observed in the detail of XRD patterns between 38–42°. Also, the doping leads to a slight increase in the width of the peaks (more important for Co^2+^ doping). So, according to the Debye–Scherrer relation the average crystallite size decrease in order: ZnO (19.4 nm), ZnO+1%Mg^2+^ (18.94 nm), ZnO+1%Co^2+^ (15.65 nm); there sizes are in correlation with ionic radius which decrease in the series: Zn^2+^–0.74 Å > Mg^2+^–0.72 Å > Co^2+^–0.70 Å.

**Figure 2:**
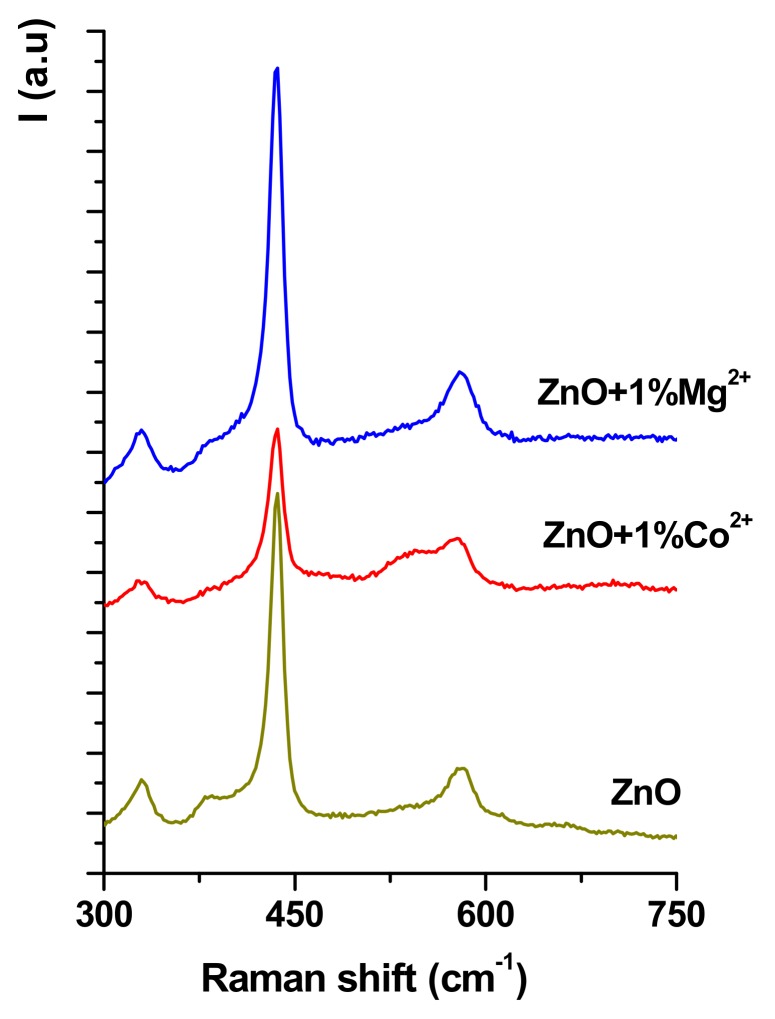
Raman spectra for undoped and Co^2+^/Mg^2+−^doped ZnO films coatings on grade TiAlV alloy substrate thermally treated at 600 °C for 2 h.

Also, the influence of the Co^2+^/Mg^2+^ doping on ZnO structure was assessed by Raman spectroscopy (Figure 2) and it can be seen that around 335, 440 and 580 cm^−1^, vibrational bands attributed to ZnO clearly emerge for all cases. By Co^2+^/Mg^2+^doping of ZnO these vibrational bands decrease or disappear with appearing new bands: for Co^2+^ doping at approx. 540 cm^−1^ and 382 cm^−1^ (shoulder), and for Mg^2+^ at 505 cm^−1^ with very lower intensity. Once again, these results demonstrate the Co^2+^/Mg^2+^ doping of ZnO.

**Figure 3:**
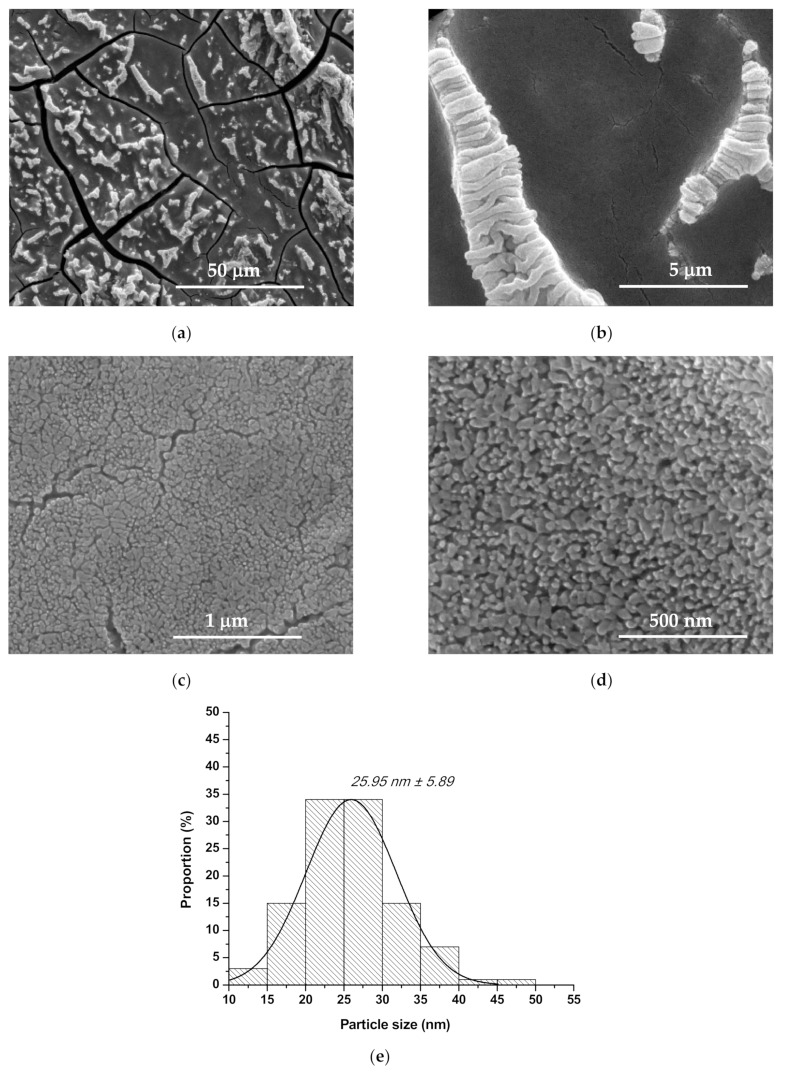
Scanning electron microscope (SEM) images (**a**–**d**) and grain size distribution (**e**) for ZnO coating.

**Figure 4:**
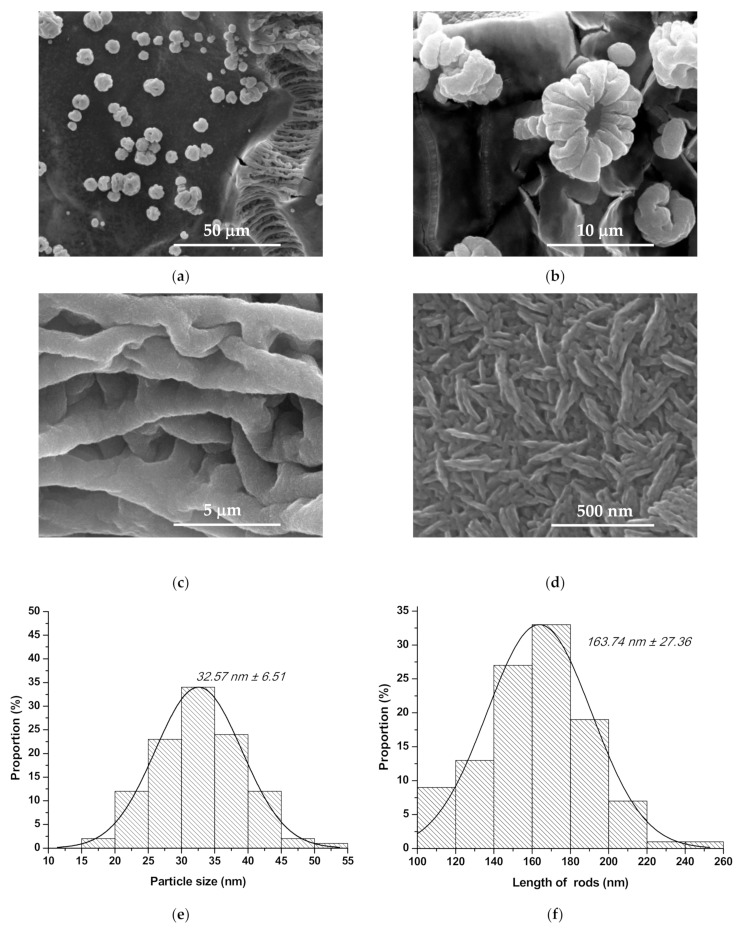
SEM images (**a**–**d**) and grain size distribution ((**e**)—quasi-spherical particles; (**f**)—rods shape agglomerates) for Co^2+^ doped ZnO coating.

**Figure 5:**
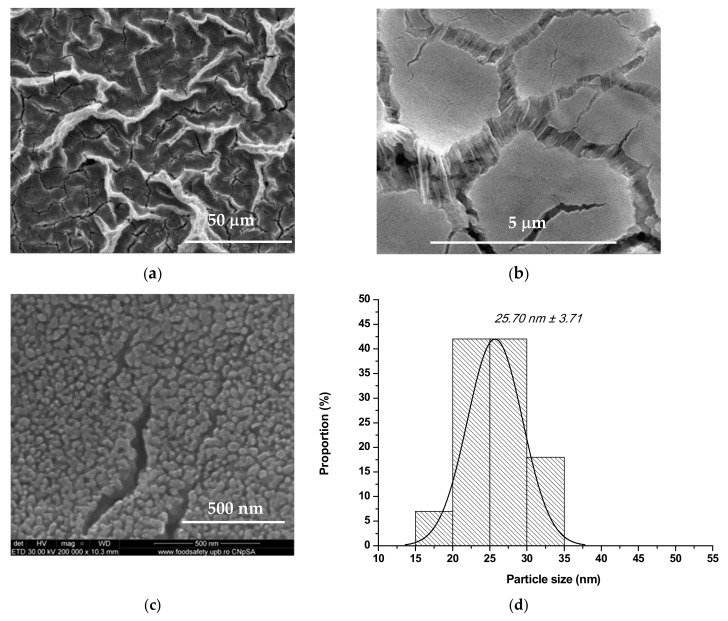
SEM images (**a**–**c**) and grain size distribution (**d**) for Mg^2+^-doped ZnO coating.

From the microstructural and particle size point of view, the obtained coatings were assessed by scanning electron microscopy (SEM) and statistical processing of those—Figure 3, Figure 4 and Figure 5. The SEM images reveal that the grain sizes and depositions morphologies depend on nature of the doping ion. Therefore, one can observe for all deposited samples, doped or not, that the particles have quasi-spherical morphology and the coatings are relatively homogenous, with columnar development. It needs to be mentioned the fact that the presence of Co^2+^ induce a morphology modification: the cvasi-spherical particles form rod like aggregates—the rod length is approx. 164 nm (Figure 4d), probably due to the difference between ionic radius of Zn^2+^ and Co^2+^ and to the smaller crystallite size in the case of Co^2+^ doping.

From Figure 3e, Figure 4e and Figure 5d one can observe the influence of ionic radius on the particle size distribution: the particles’ average size is approximately 26 nm in the case of undoped ZnO and Mg^2+^-doped ZnO, and approximately 32 nm in the case of Co^2+^ doped ZnO; although all particle size distributions are monomodal, it must be pointed out the fact that Mg^2+^-doped ZnO registered the most narrow distribution of approximately 80% from quasi-spherical particles are between 20 and 30 nm.

Also, all depositions present agglomerations in the form of folds or flowers, probably due to the changing the drying pressure and some gelifing zones formed under the action of the centrifugal force during spin-coating deposition, which prevents the uniform/flat spreading of the coating [31,32,33]. There are cracks in the layers, which can be explained by the evaporation process of the solvent, but which do not constitute a disadvantage for the proposed use.

### 3.2. ZnO and Co^2+^/Mg^2+^-Doped ZnO Depositions in In Vitro Investigation

**Figure 6:**
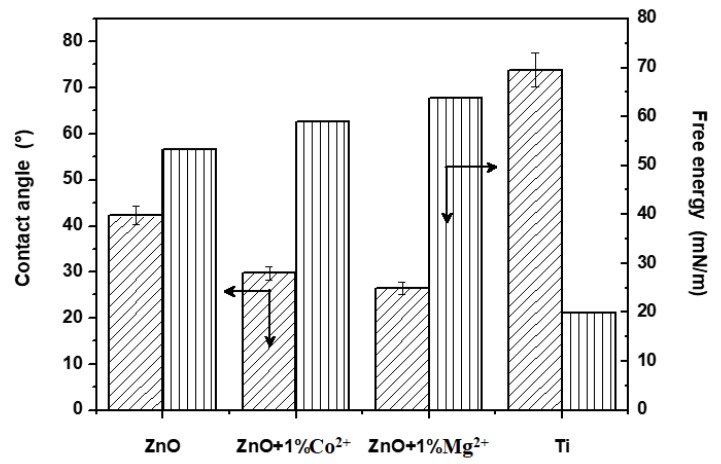
The contact angle and free energy for TiAlV alloy substrate and undoped and Co^2+^/Mg^2+^ doped ZnO films coatings on that, at thermally treated at 600 °C for 2 h.

The hydrophilic behaviour influences the biocompatibility of materials and the cellular adherence on their surface. To assess the hydrophilic behaviour, the contact angle and free energy were determined (Figure 6), and the results display all the coatings, as compared to titanium substrate (with higher contact angle values and lower free energy value). Therefore, the deposited samples hydrophilicity decreases in order ZnO+1%Mg^2+^ > ZnO+1%Co^2+^ > ZnO. These results are sustained by increased basicity of Mg^2+^ doped ZnO (the presence of a more basic ion increases the sample hydrophilicity) and by the narrow particle size distribution.

**Figure 7:**
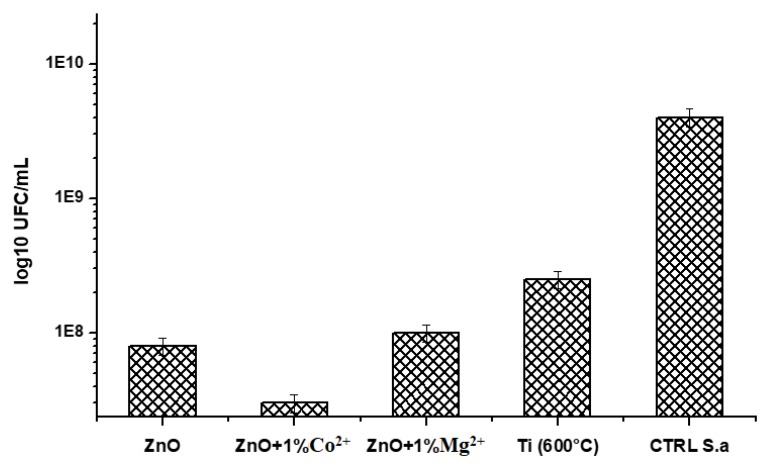
The *Staphylococcus aureus (S. aureus)* biofilm development inhibition in the presence of undoped and Co^2+^/Mg^2+^-doped ZnO film coatings on TiAlV alloy substrate.

Figure 7 shows that the all ZnO films deposited by the spin coating technique exhibit a very good inhibition of *Staphylococcus aureus* (*S. aureus*) biofilm development, with the formed colonies per unit of mL (CFU/mL) being smaller than those formed on uncoated Ti. These results are sustained by literature data [2,12,24,25,34,35]. The more obviously inhibition of Co^2+^-doped ZnO is probably due to sample morphology.

**Figure 8:**
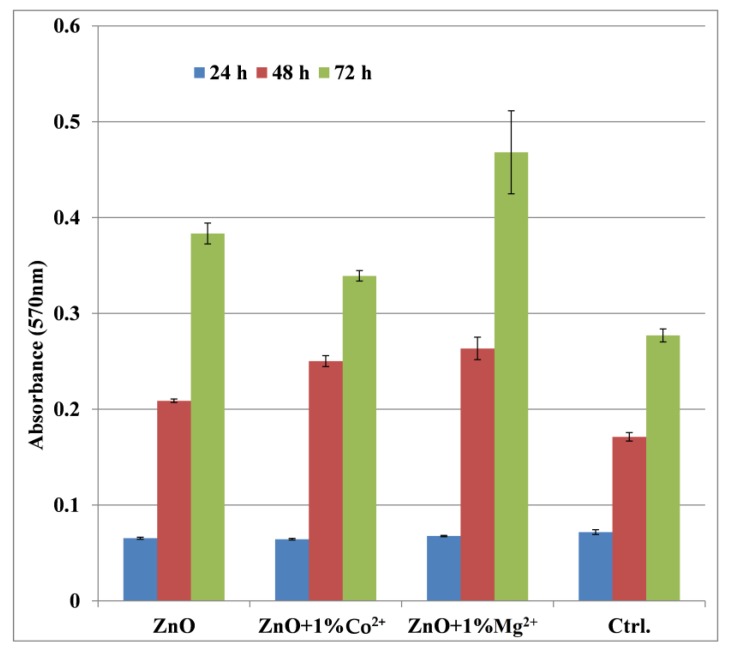
The MTT assay on undoped and Co^2+^/Mg^2+^-doped ZnO films coatings on TiAlV alloy substrate.

**Figure 9:**
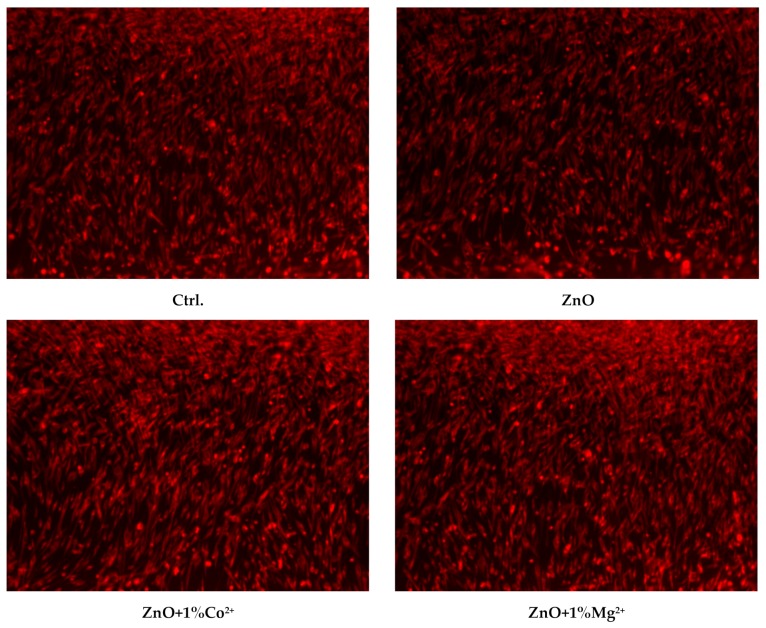
The fluorescence microscopy images performed on undoped and Co^2+^/Mg^2+^ doped ZnO films coatings on TiAlV alloy substrate.

**Figure 10:**
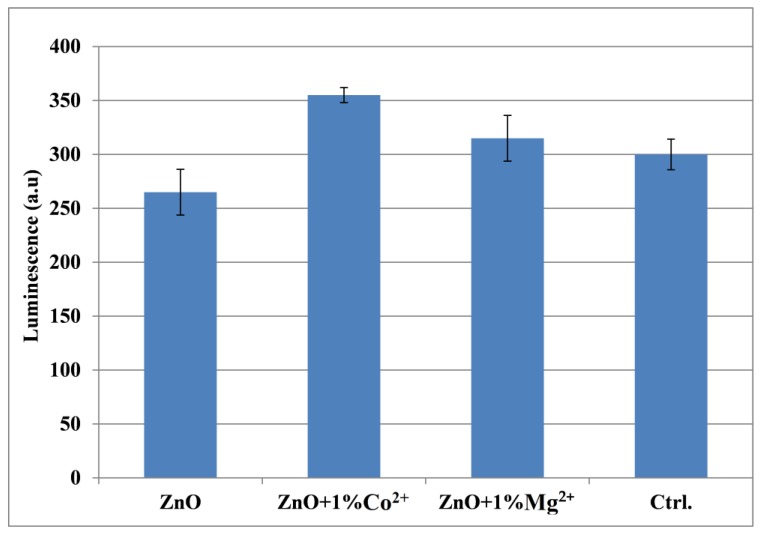
The glutathione (GSH) assay on undoped and Co^2+^/Mg^2+^-doped ZnO film coatings on TiAlV alloy substrate.

Also, as it is known from the literature that ZnO in the form of nanoparticles has a toxic effect on cells over a certain concentration [34], so cell viability and proliferation must be assessed on the deposited samples. Therefore, the film coatings were in vitro characterized by carrying out cell tests in which the viability and cell proliferation were monitored. Thus, Figure 8 presents the cell proliferation assay—MTT, associated with fluorescence microscopy—and Figure 9 and Figure 10 the oxidative stress caused by these film coatings on AFSCs (mesenchymal stem cells isolated from amniotic fluid).

From Figure 8 it is observed that the ZnO film coating had no cytotoxic effect on the cells, unlike ZnO nanoparticles, the absorption values being higher than the control sample. At 24 h there is a relatively equal proliferation for all 3 film coatings and control sample; following that, at 48 h, the proliferation of AFSCs will be stimulated, and the absorbance values will exceed the control sample. Finally, at 72 h, all the film coatings stimulated the metabolism of the cells, therefore one can observe a more significant creation and proliferation of cells as compared with control sample. The highest value of absorbance after 72 h it is observed at Mg^2+^ doped ZnO, followed by undoped ZnO and Co^2+^-doped ZnO. These results are in correlation with the samples hydrophilicity (the Mg^2+^-doped ZnO presents the lowest value for the contact angle).

The fluorescence microscopy images from Figure 9 prove that the cells are viable, the film coatings have no cytotoxic effect on them, thus confirming the biochemical results. No dead cells or cell debris are observed, with AFSCs having normal morphology with fibroblast-like appearance. Cellular metabolism is active, as proven by fluorescence images, the cells with fluorescent dye incorporated into the cytoplasm being viable. As compared with the control sample, it should be mentioned the fact that doped films present similar behaviour, due to their higher hydrophilicity.

Regarding the oxidative stress tests, one can see that all the film coatings are within the limit of 15% as compared with the control sample, producing oxidative stress on the cells slightly higher than the control in the case of Co^2+^/Mg^2+^-doped ZnO coating and smaller than this for undoped ZnO coating—Figure 10. Therefore, cellular tests pointed out the fact that the film coatings obtained can be cell-friendly supports.

## 4. Conclusions

Due to the fact that ZnO is one of the most interesting and promising oxide nanomaterials used in implantology at the moment, in this paper the influence of ZnO doping with Co^2+^ and Mg^2+^ ions was investigated in order to obtain it and to deposit coatings on titanium alloy substrate through the spin coating method. These coatings are useful for a better osseointegration of a titanium alloy implant and for inducing antibacterial behaviour.

For this purpose, three solutions of ZnO undoped and doped with Co^2+^ and Mg^2+^ were prepared by the sol-gel method and deposited using the spin coating installation on a metallic substrate, and different characterization techniques were used on the coatings obtained: XRD, Raman spectroscopy, SEM, determination of specific properties, antibacterial and cellular tests.

Following the analysis results, the successful incorporation of Co^2+^ and Mg^2+^ ions into the wurtzite network of ZnO was demonstrated, with a decrease in its crystallite size, which is attributed to the smaller ionic radius of the two dopants.

Also, from the microstructural point of view, the wurtzite-like structure of ZnO was noticed. It should be mentioned that the presence of cobalt and magnesium did not induce the formation of other phases, but only a decrease of the particle size attributed, again, to the ion radius.

Following the specific properties results, it is necessary to point out that the coating with undoped and doped ZnO increases the hydrophilicity of the titanium substrate, thus improving the biocompatibility and cell adhesion to the substrate.

In addition, the tests demonstrated the antibacterial behaviour of the three film coatings, and from the cellular tests it can be concluded that the coatings obtained stimulated the cell proliferation and did not exhibit toxic effects on the cells.

Thus, it can be said that the coatings of zinc oxide (undoped or doped with Co^2+^ and Mg^2+^) can improve the antibacterial properties of the substrate on which they are deposited, which makes this method a good alternative for increasing the antibacterial activity of the metallic materials used in implantology.
